# Student perceptions of a tutorial-based model for early clinical-humanities integration: a cross-sectional study

**DOI:** 10.3389/fmed.2026.1860084

**Published:** 2026-06-16

**Authors:** Yan Wu, Ji Shu, Xiaoqing Li, Yixin Zhang, Yuhang Zhu, Wen Li, Xiangrong Xu, Jingyi Li

**Affiliations:** Women’s Hospital, School of Medicine, Zhejiang University, Hangzhou, China

**Keywords:** clinical-humanities integration, cross-sectional evaluation, early-stage medical students, medical education, tutorial-based model

## Abstract

**Background:**

The integration of clinical skills and humanistic literacy is a paramount objective in modern medical education. This study evaluates a novel tutorial-based model designed to promote early clinical-humanities integration for first-year medical students.

**Methods:**

A cross-sectional study was conducted at Zhejiang University School of Medicine. A self-reported questionnaire was administered to first-year medical students enrolled in the “Whole Life Cycle Health I” course (*N* = 275). The questionnaire assessed their adaptation to this model, learning interest, perceived promotion of clinical-humanities integration, module effectiveness, and self-rated competency improvement.

**Results:**

Most students reported good adaptation (90.9%) and increased learning interest (88.0%). Although adaptation levels were comparable between genders, male students expressed significantly stronger agreement regarding enhanced interest and the model’s effectiveness in fostering integration (*p* < 0.05). Key practical modules—including “Simulated Outpatient Role-Playing,” “AI-Powered Science Video Production,” and “Postpartum Home Visits”—were rated as highly effective. Students reported substantial improvement in patient communication (87.3%) and teamwork (85.1%), despite challenges like time constraints and task integration difficulties.

**Conclusion:**

The tutorial-based model is perceived by students as a feasible, promising, and well-received strategy for early clinical-humanities integration, demonstrating significant potential for broader implementation in medical education reform.

## 1 Introduction

The fundamental goal of medical education is to cultivate physicians with both excellent clinical skills and profound humanistic literacy. However, traditional models often compartmentalize these domains, creating a rift between clinical practice and humanistic care in curricula (1). This divide hinders the integration of humanistic principles into clinical practice, potentially eroding interpersonal skills and compromising healthcare quality, as suggested by declining patient satisfaction with physician experience (2, 3). To address this divide, medical educators worldwide are exploring innovative teaching models that facilitate the early integration of clinical skills and humanities. Student-centered approaches such as Problem-Based Learning (PBL) and Case-Based Learning (CBL) show particular promise. These methods enhance students’ humanistic literacy and clinical skills by encouraging active discussion and reflection (4). Furthermore, transforming the learning environment to engage students as active participants rather than passive observers—fostering self-directed learning, critical thinking, and teamwork in authentic or simulated clinical settings—provides an ideal platform for developing both clinical reasoning and humanistic judgment (5). Although these pedagogical principles are widely recognized, empirical research on their systematic application in clinical-humanities integration courses for early-stage medical students remains limited. How to effectively stimulate student interest, investigate feedback from different student groups, and simultaneously shape their clinical abilities and humanistic literacy at the outset of medical education remains an underexplored area of research.

The tutorial method, a student-centered approach emphasizing small-group interaction and participatory learning, occupies an important niche in medical education. It is widely employed in clinical skills training—for instance, in foundational laparoscopic surgery instruction, where small-group discussions and hands-on practice enhance students’ theoretical understanding and technical proficiency (6). In clinical teaching, tutorials serve as a core method for knowledge construction, helping students develop complex thinking and diagnostic skills through progressive exposure to real cases (7). Within humanities education, the tutorial method particularly emphasizes communication skills, ethical awareness, and empathy (8, 9). For instance, in the Italian medical education system, it is advocated as an alternative to the traditional teacher-centered model, aiming to cultivate students’ ethical awareness and communication skills to better address health issues in modern society (10). Overall, the tutorial method enhances medical education quality and strengthens the integration of humanistic care into practice by promoting self-directed learning and critical thinking.

Although the tutorial approach is well-established, most existing studies focus on senior students or isolated teaching applications. Research remains scarce on how to effectively leverage a series of carefully designed tutorial activities to stimulate interest, examine differential responses to various instructional components, and simultaneously develop clinical abilities and humanistic literacy from the very beginning of medical education. This study employs a cross-sectional survey to systematically evaluate the implementation of the tutorial-based model in the “Whole Life Cycle Health I” course from the student perspective. It focuses on acceptability, effectiveness in promoting clinical-humanities integration, impact on core competencies, and the presence of gender differences and challenges. The findings aim to provide preliminary evidence and practical insights to inform curriculum reform, promote personalized teaching, and disseminate this student-centered, practice-oriented model.

## 2 Materials and methods

### 2.1 Study design and course context

This study was a course-based cross-sectional survey conducted in June 2025 at Zhejiang University School of Medicine (Hangzhou, China). It centered on the “Whole Life Cycle Health I” course, a newly developed innovation for first-year undergraduates targeting early clinical-humanities integration. This course spanned 16 weeks (covering the Spring and Summer semesters of 2025), with a scheduled 3-h tutorial or practical session held every 2 weeks. Additionally, students were required to attend three lectures totaling 8 h, covering fundamental concepts in obstetrics and gynecology as well as medical humanities. The course shifted the instructional focus from traditional lectures to student-centered practice, aiming to foster medical students’ clinical abilities and humanistic literacy synergistically.

### 2.2 Participants

This study included first-year undergraduate medical students enrolled in the “Whole Life Cycle Health I” course during the 2024–2025 spring–summer semester. In the final class session, a structured, self-reported online questionnaire was distributed to all students via the web-based survey tool Sojump (Changsha ran Xing InfoTech Ltd., China). Informed consent was obtained from the students, and the questionnaire was anonymous and voluntary.

### 2.3 Course design and teaching methods

The study focused on the first-year compulsory course “Whole Life Cycle Health I.” This integrated course, encompassing the beginning of life and the processes of pregnancy, childbirth, and parenting, aims to develop students’ interdisciplinary integration skills, humanistic care awareness, and proactive learning habits through early clinical exposure. To effectively integrate humanities and clinical practice, the course adopted a tutorial-based model as its core teaching method, characterized by the following features:

(1) Small-group practice and discussion: students were divided into fixed groups of 3–4. Under the guidance of a tutor, each group completed a series of tasks. During the tasks, students were required to think independently and express their insights, while the tutor provided necessary guidance and feedback to promote students’ self-directed learning and teamwork skills.
(2) Diversified modules: the course featured a variety of practical modules designed to bridge theory and practice. The core modules immersed students in realistic scenarios: “Simulated Outpatient Role-Playing” allowed students to practice doctor-patient communication and navigate ethical dilemmas; “AI-Powered Science Video Production” tasked them with creating accurate yet accessible health education content using digital tools; and “Postpartum Home Visits” provided firsthand experience in conducting assessments and understanding the psychosocial context of patients’ lives. Optional modules, such as the “Hospital Exploration Journey,” “Pregnancy Guard Workshop,” “Smart Medical Decoding Room,” and “Holographic Birth Witness,” further enriched their perspective. Together, these modules were designed to cultivate students’ problem-solving, communication, and reflective practice abilities.
(3) Interdisciplinary integration: each task required students to analyze and make decisions using preliminary clinical knowledge and humanistic-social science perspectives. For example, in the “Postpartum Home Visits” module, students were asked to assess not only the mother’s physical recovery (clinical) but also her emotional well-being, family support system, and access to social services (humanistic-social). They then reflected on how social determinants such as income, housing, and childcare responsibilities might influence postpartum health outcomes.

### 2.4 Questionnaire development

The self-report questionnaire was developed through a multi-step process. First, an initial item pool was generated based on course learning objectives (e.g., adaptation to active learning, integration of clinical and humanistic skills, self-rated competency improvement). Second, a panel of five experts—two medical education researchers, two clinical faculty, and one medical humanities scholar—reviewed the items for content validity, clarity, and relevance. Third, the questionnaire was piloted with 30 first-year medical students, feedback was used to refine wording and reduce ambiguity. The final 16-item questionnaire covered demographics (age, gender), 8 single-choice questions (including 6 using a 5-point Likert scale), 5 multiple-choice questions, and 1 open-ended question (shown as Supplementary file 1). The 6 Likert-scale items formed a subscale assessing perceptions of the tutorial model and its effectiveness in promoting clinical-humanities integration; this subscale showed good internal consistency (Cronbach’s *α* = 0.887).

### 2.5 Data collection

The anonymous self-report questionnaire was distributed via the web-based survey tool Sojump during the final class. Participants completed the questionnaire in approximately 3 min on average. The questionnaire included items on: basic Information (2 items), overall evaluation of the tutorial-based model (4 items), evaluation of course module effectiveness (6 items), skills enhancement and future suggestions (4 items). The open-ended question invited free-text suggestions.

### 2.6 Statistical analysis

Data analysis was performed using the Statistical Package for Social Sciences, version 26 for Windows, IBM Corp., Armonk, NY, USA. Variables were summarized as mean ± standard deviation (SD) or frequency (%), as appropriate. Gender differences were assessed using Chi-square or Mann–Whitney *U* tests, with statistical significance set at *p* < 0.05. Open-ended suggestions underwent inductive thematic analysis to identify prominent themes.

## 3 Results

### 3.1 Sample characteristics

Among all students who enrolled in the course, a total of 276 completed the questionnaire survey. After excluding one incomplete response, data from 275 students were ultimately included in the analysis. The sample consisted of 145 males and 130 females, with a mean age of 18.82 ± 0.81 years (males: 18.81 ± 0.82; females: 18.84 ± 0.81).

### 3.2 Student adaptation and acceptance

The survey revealed that students reported good adaptation and high acceptance of the tutorial method. 90.9% of students reported being “Very well adapted” or “Relatively well adapted” to this tutorial-based model. When asked to compare the tutorial-based model in this course with their experiences in other courses that used entirely lecture-based teaching, 88.0% of students believed that the tutorial-based model was more effective in enhancing their learning interest. Further analysis found that a significantly higher proportion of male students reported increased learning interest compared to female students (“Significantly enhanced” 36.5% vs. 20.8%; “Somewhat enhanced” 53.1% vs. 65.4%, *p* < 0.05). Although overall adaptation levels did not differ significantly by gender, more males (33.1%) than females (26.1%) selected “Very well adapted.” Most students (88.4%) supported extending the method to other medical courses, indicating broad acceptance and potential for dissemination (Table 1).

**Table 1:** Students’ overall evaluation of the tutorial-based model (*N* = 275).

Items		Male (*N* = 145)	Female (*N* = 130)	*p*-value	Total (*N* = 275)
How well did you adapt to the tutorial-based model used in this course?	Very well adapted	48 (33.1%)	34 (26.1%)	NS*	82 (29.8%)
	Relatively well adapted	86 (59.3%)	82 (63.1%)		168 (61.1%)
	Neutral	9 (6.2%)	13 (10.0%)		22 (8.0%)
	Poorly adapted	2 (1.4%)	1 (0.8%)		3 (1.1%)
	Not adapted at all	0 (0.0%)	0 (0.0%)		0 (0.0%)
Compared with other courses that used entirely lecture-based teaching, did the tutorial-based model in this course enhance your learning interest more?	Significantly enhanced	53 (36.5%)	27 (20.8%)	<0.01*	80 (29.1%)
	Somewhat enhanced	77 (53.1%)	85 (65.4%)		162 (58.9%)
	No difference	11 (7.6%)	16 (12.3%)		27 (9.8%)
	Somewhat decreased	3 (2.1%)	2 (1.5%)		5 (1.8%)
	Significantly decreased	1 (0.7%)	0 (0.0%)		1 (0.4%)
How effective do you think the tutorial-based model was in promoting the integration of “clinical skills” and “humanistic care”?	Very effective	72 (49.7%)	41 (31.5%)	<0.01*	113 (41.1%)
	Moderately effective	63 (43.4%)	77 (59.2%)		140 (50.9%)
	Neutral	6 (4.1%)	11 (8.5%)		17 (6.2%)
	Limited effectiveness	4 (2.8%)	1 (0.8%)		5 (1.8%)
	Not effective at all	0 (0.0%)	0 (0.0%)		0 (0.0%)
Do you think the tutorial-based model is worth applying and promoting in other medical courses?	Yes	128 (88.3%)	115 (88.5%)	NS^#^	243 (88.4%)
	No	17 (11.7%)	15 (11.5%)		32 (11.6%)

*Mann–Whitney *U* test is used here; ^#^Chi-square test is used here.

### 3.3 Effectiveness in promoting clinical-humanities integration

**Figure 1:**
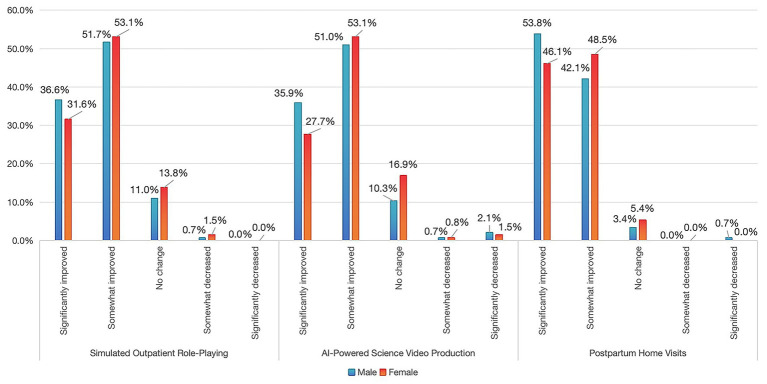
Degree of perceived helpfulness of different core practical modules for understanding clinical-humanities integration.

The course’s primary goal—early integration of clinical skills and humanistic care—was perceived by students as met effectively: 92.0% of students rated the tutorial method “very” or “moderately” effective, with significantly higher approval among males (*p* < 0.05; Table 1). Specific activities were also highly rated: “Simulated Outpatient Role-Playing” deepened understanding of ethical and humanistic issues in doctor-patient communication (86.5%); “AI-Powered Science Video Production” improved integration of medical knowledge and humanistic expression (84.0%); and “Postpartum Home Visits” heightened attention to social and psychological needs (95.3%). Although gender differences in perceived helpfulness of the three core modules were not statistically significant, a greater proportion of males rated each as “Significantly improved” (Figure 1).

Among optional modules, the “Pregnancy Guard Workshop” (93.7%) and “Hospital Exploration Journey” (91.5%) were deemed most beneficial for understanding integration (Table 2). Module preferences differed by gender: males favored the “Hospital Exploration Journey” and “Smart Medical Decoding Room,” whereas females preferred the “Holographic Birth Witness” and “Pregnancy Guard Workshop.” In evaluations, males rated the “Hospital Exploration Journey” significantly more helpful than females did (*p* < 0.05); no other significant gender differences emerged. These findings indicate that, from the students’ perspective, practice-oriented teaching activities are an effective path to achieving clinical-humanities integration.

**Table 2:** Students’ evaluation and preferences for the elective practice modules.

Items	Do you think it is helpful in understanding the integration of clinical and humanities aspects?	Male	Female	*p*-value	Total (*N* = 275)
Hospital exploration journey	Yes	42 (97.7%)	23 (82.1%)	0.03	65 (91.5%)
	No	1 (2.3%)	5 (17.9%)		6 (8.5%)
Pregnancy guard workshop	Yes	26 (89.7%)	33 (97.1%)	NS	59 (93.7%)
	No	3 (10.3%)	1 (2.9%)		4 (6.3%)
Smart medical decoding room	Yes	38 (86.4%)	19 (82.6%)	NS	57 (85.1%)
	No	6 (13.6%)	4 (17.4%)		10 (14.9%)
Holographic birth witness	Yes	25 (86.2%)	39 (88.6%)	NS	64 (87.7%)
	No	4 (13.8%)	5 (11.4%)		9 (12.3%)

Fisher’s exact test is used here.

### 3.4 Improvement in core competencies

**Figure 2:**
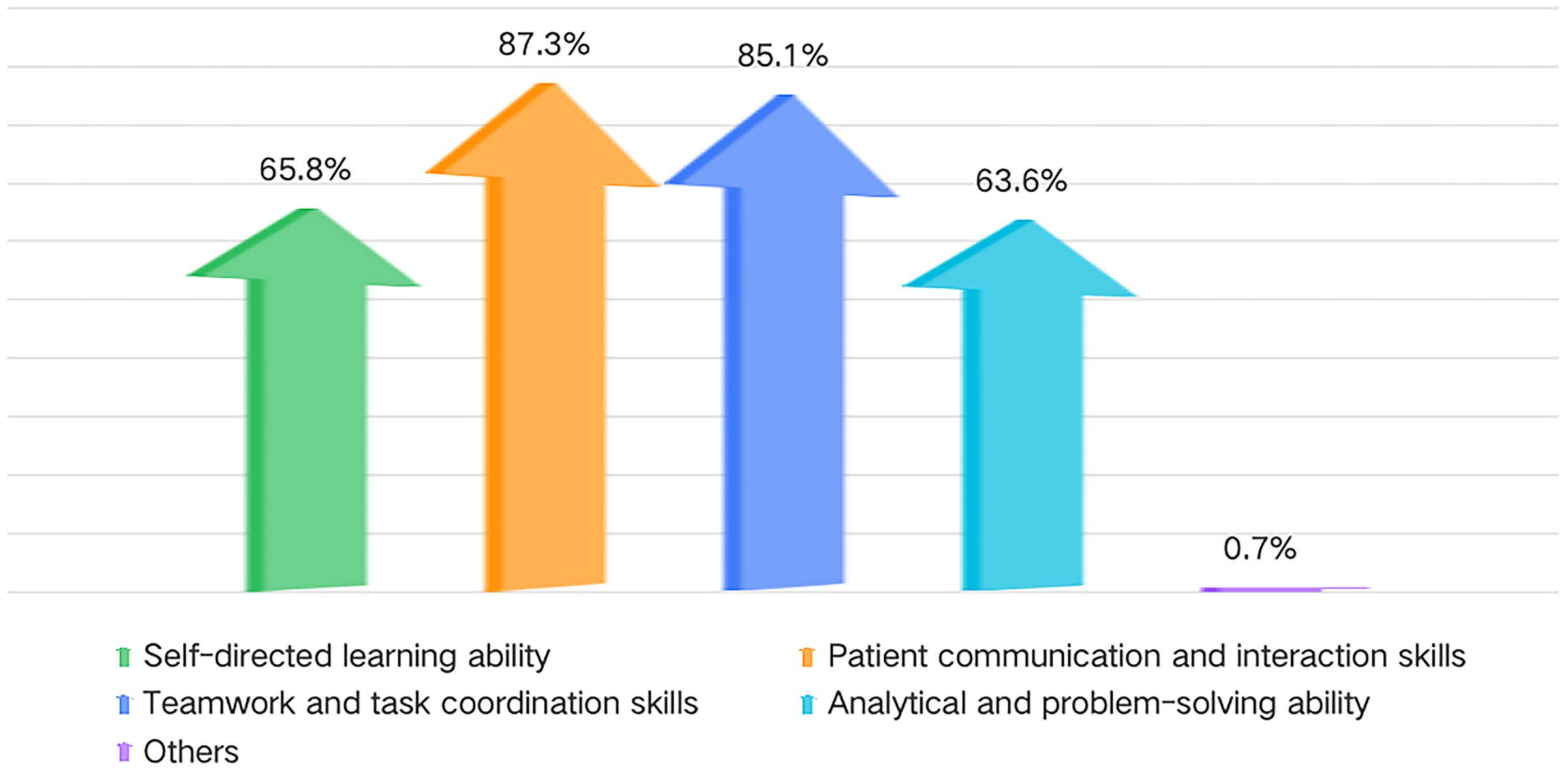
Student self-rated competency improvement (multiple-choice, *N* = 275).

Students reported that the course enhanced their comprehensive abilities (Figure 2). The most marked gains were in “Patient communication and interaction skills” (87.3%) and “Teamwork and task coordination skills” (85.1%), aligning with the tutorial method’s emphasis on small-group discussion and collaboration. Significant progress was also reported in “Self-directed learning ability” (65.8%) and “Analytical and problem-solving ability” (63.6%). No significant gender differences were observed in competency improvements.

### 3.5 Challenges and suggestions for improvement

Despite overall positive feedback, students endorsed several challenges, primarily “Limited time for practical sessions” (65.8%) and “Difficulty connecting humanistic topics with clinical tasks” (51.3%). Gender responses were similar overall. During home visits, “Establishing a trusting relationship with the family” (77.5%) and “Administering the questionnaire (e.g., sensitivity of questions)” (77.1%) were identified as the most challenging aspects. Although not statistically significant, more males than females found “Establishing a trusting relationship with the family” (81.4% vs. 73.1%) and “Communication during the informed consent process” (40.7% vs. 30.0%) challenging.

Students endorsed incorporating more diverse teaching formats. The most desired were “Gamified learning (e.g., medical knowledge challenges, scenario simulation games)” (58.5%), “Blended online and offline teaching” (57.1%), and “Immersive virtual simulation experiences (e.g., interaction with AI patients)” (50.9%). A higher proportion of males preferred gamified learning (63.4% vs. 53.1%), though this difference was not significant. Open-ended recommendations emphasized increasing practical hours and real-case exposure, improving pre-visit training and preparation, and optimizing guidance on AI tools.

## 4 Discussion

This cross-sectional survey systematically evaluated a tutorial-based model in an early clinical-humanities integration course for junior medical students. Key findings indicate that this student-centered, practice-oriented approach was not only well-received but also perceived by students as effective in fostering integration and enhancing core competencies. Notably, gender-specific response patterns emerged, offering valuable insights for personalized instructional design.

High student acceptance and satisfaction were evident, with over 90% adaptation rates and strong support for broader implementation. This positive reception may be attributed to two factors. First, the tutorial model accurately responds to the inherent needs of the new generation of “digital native” medical students for interactivity, participation, and learning autonomy (11), which is consistent with the positive feedback from medical undergraduates regarding interactive learning methods like “teaching seminars” in the ECG teaching process (12). Second, the course design materialized abstract humanistic concepts into executable tasks such as “Simulated Outpatient Role-Playing” and “Postpartum Home Visits,” building a scaffold from theory to practice for junior students and effectively reducing anxiety associated with early exposure to clinical scenarios, aligning with the observed enhancements in group learning and problem-solving abilities in simulation tutorials (13). Although there was no statistical difference in the level of adaptation between male and female students, a higher proportion of males selected “very well adapted” and more male students believed the model enhanced their learning interest. This suggests that male students may respond more positively to the interactive, problem-solving-oriented learning environment created by the tutorial format.

Comparison with existing active learning models: The high acceptance and perceived effectiveness of our tutorial-based model are consistent with previous reports on student-centered approaches such as PBL and CBL (4). However, unlike PBL which primarily emphasizes diagnostic reasoning through clinical cases, our model uniquely integrates clinical skills with humanities through experiential modules (e.g., home visits, role-playing). Furthermore, while most PBL/CBL studies focus on senior students, our findings demonstrate that even first-year undergraduates can benefit from early exposure to this integrated, practice-oriented format.

Students widely perceived that clinical-humanities integration can be achieved not only through theoretical instruction but also via experiential learning in well-designed practical activities. The majority of students (92.0%) regarded the tutorial model as effective for integration. “Simulated Outpatient Role-Playing” provided a safe environment to rehearse and reflect on ethical dilemmas in doctor-patient communication, reinforcing empathy and communication skills—consistent with effects observed with virtual and mixed reality simulations (14). The “AI-Powered Science Video Production” task compelled students to “translate” specialized terms into lay language, constituting a deep exercise in knowledge integration and humanistic expression, analogous to using AI tools for personalized learning resource design (15). Most importantly, “Postpartum Home Visits” extended learning beyond the classroom into real-family contexts, exposing students to social determinants of health and underscoring that humanistic care is not merely an attitude but a core clinical skill for understanding illness contexts and building trust—resonating with the interactive and reflective dimensions highlighted in professional identity formation (16). This model of “Learning by doing, integrating through reflection” was regarded by students as an effective path to achieving the course’s core objectives. Gender differences emerged in module preferences and perceived challenges: males leaned toward technology-exploration modules, while females favored life-process and care-oriented modules. Males also reported greater difficulty with soft-skill tasks like “Establishing trust relationships” during home visits. These patterns likely reflect socio-culturally influenced interests and confidence levels, suggesting that diversified module options and targeted skill support could enhance future curriculum design.

Among student self-rated competency improvements, “Patient communication and interaction skills” (87.3%) and “Teamwork and task coordination skills” (85.1%) were the most prominent, with no significant gender differences in the extent of improvement. This directly confirms the inherent advantage of the tutorial-based model in cultivating soft skills and is supported by evaluations of mixed reality tutorials (17). Challenges, however, were instructive. “Limited time for practical sessions” and “Difficulty connecting humanistic topics with clinical tasks” indicated a need for more supported, deep interdisciplinary reflection under high-intensity workloads, echoing technical and administrative hurdles noted in e-tutorial training (18). Future course designs should therefore prioritize process optimization, efficient guidance tools, and structured reflection frameworks over simply adding activities. Importantly, challenges like “building trust” and “addressing sensitive issues” during home visits represent authentic, central humanistic dilemmas in medical practice; openly discussing these in class constitutes valuable learning itself. Strong student interest in “gamified teaching” and “virtual simulation” signals promising directions for innovation. The trend toward male preference for gamification, along with emerging technologies like AI virtual standardized patients, could effectively supplement teaching resources and allow repetitive practice in high-risk scenarios, aligning with developments in virtual reality and serious games for medical education (14, 19). Implementing these technologies could benefit from considering gender-specific preferences to maximize educational impact.

This study has several limitations. First, it is a single-center study, and the generalizability of the results needs to be validated cautiously. Second, the absence of a control group prevents direct comparison with traditional teaching methods and precludes establishing clear causal effects. Third, the use of self-report questionnaires means results may be subject to social desirability bias. Furthermore, the exploration of gender differences in this study is preliminary, and the underlying reasons (such as socio-cultural factors, personality traits, etc.) need to be further revealed by subsequent qualitative research. Future studies should combine objective assessment methods (such as standardized scoring of role-play videos), in-depth interviews, and long-term follow-up to more comprehensively evaluate the impact of this teaching method and the complex individual difference factors involved.

In conclusion, this study provides preliminary empirical insights that suggest the tutorial-based model may be a strategy perceived by students as effective for early clinical-humanities integration. Based on student feedback, this model successfully engaged junior medical students, and students reported perceived improvements in their core competencies, laying a solid foundation for their development as clinically skilled and humanistically grounded physicians. However, these conclusions must be interpreted with caution. Future studies should incorporate objective assessment tools and controlled designs to deepen task design, bolster process guidance, accommodate individual learner characteristics, and further explore technology-teaching integration to continuously advance medical education quality.

## Data Availability

The raw data supporting the conclusions of this article will be made available by the authors, without undue reservation.
