# Supplementary material for: Student perceptions of a tutorial-based model for early clinical-humanities integration: a cross-sectional study
Source: Front Med (Lausanne). 2026 Jun 16;13:1860084. doi: 10.3389/fmed.2026.1860084 (PMC13314468; doi:10.3389/fmed.2026.1860084)
Supplement: Supplementary file 1 [file Table_1.DOCX]

**Questionnaire on the Effectiveness of the Tutorial-based Model in Early Clinical-Humanities Integration**

Dear Student,
In order to optimize the medical education model and promote the deep integration of clinical skills and humanistic literacy, we sincerely invite you to participate in this anonymous survey. Your feedback will provide an important basis for the improvement of this project. Thank you for your support!

I. Basic Information

1. Your Age: ________ years

2. Your Gender: ○ Male ○ Female

II. Overall Evaluation of the Tutorial-based Model

3. How well did you adapt to the tutorial-based model used in this course?
○ Very well adapted ○ Relatively well adapted ○ Neutral ○ Poorly adapted ○ Not adapted at all

1. Compared with other courses that used entirely lecture-based teaching, did the tutorial-based model in this course enhance your learning interest more?
   ○ Significantly enhanced ○ Somewhat enhanced ○ No difference ○ Somewhat decreased ○ Significantly decreased
2. How effective do you think the tutorial-based model was in promoting the integration of "clinical skills" and "humanistic care"?
   ○ Very effective ○ Moderately effective ○ Neutral ○ Limited effectiveness ○ Not effective at all
3. Do you think the tutorial-based model is worth applying and promoting in other medical courses?
   ○ Yes ○ No

III. Evaluation of Course Module Effectiveness

7. Which specific optional practical module did you participate in?
□ Hospital Exploration Journey
□ Pregnancy Guard Workshop
□ Smart Medical Decoding Room
□ Holographic Birth Witness

□ Did not participate

8. Regarding the modules you have participated in, do you think it is helpful in understanding the integration of clinical and humanities aspects?"

(Please skip this question if you did not participate in any module)
○ Yes ○ No

1. Did the "Simulated Outpatient Role-Playing" in the tutorial sessions help you better understand the ethical and humanistic issues in doctor-patient communication?
   ○ Significantly improved ○ Somewhat improved ○ No change ○ Somewhat decreased ○ Significantly decreased
2. Did the task of creating a science education video using AI tools enhance your ability to integrate medical knowledge and humanistic expression?
   ○ Significantly improved ○ Somewhat improved ○ No change ○ Somewhat decreased ○ Significantly decreased
3. Did the home visit task (e.g., obtaining informed consent, completing questionnaires) make you pay more attention to the social background and psychological needs of pregnant women?
   ○ Significantly improved ○ Somewhat improved ○ No change ○ Somewhat decreased ○ Significantly decreased
4. What did you find most challenging about the “Postpartum Home Visits” task? (Multiple choice)
   □ Establishing a trusting relationship with the family
   □ Communication during the informed consent process
   □ Administering the questionnaire (e.g., sensitivity of questions)
   □ Coordinating medical advice with the family's actual needs
   □ Other (Please specify: __________)

IV. Skills Enhancement and Future Suggestions

13. Through this course, in which of the following areas did you experience significant improvement? (Multiple choice)
□ Self-directed learning ability

□ Patient communication and interaction skills
□ Teamwork and task coordination skills

□ Analytical and problem-solving ability
□ Other (e.g., critical thinking and reflective ability, etc.: __________)

14. What were the main difficulties you encountered during the course? (Multiple choice)
□ Limited time for practical sessions
□ Difficulty immersing oneself in role-playing tasks
□ Complexity of operating AI tools
□ Difficulty connecting humanistic topics with clinical tasks
□ Other (Please specify: __________)

1. What additional teaching formats or activities would you like to see added in future courses to optimize the learning experience? (Multiple choice)
   □ Gamified learning (e.g., medical knowledge challenges, scenario simulation games)
   □ Blended online and offline teaching (e.g., online case database + offline seminars)
   □ Immersive virtual simulation experiences (e.g., interaction with AI patients)
   □ Medical-humanities creative activities (e.g., scriptwriting, short film production)
   □ Other (Please specify: __________)
2. Your specific suggestions for optimizing the course design or the tutorial-based model:

________________________________________.

Thank you for your participation!

Note: This questionnaire is anonymous. Data will be used for academic research only. Please feel free to fill it out.
